# Non-ciliary Roles of IFT Proteins in Cell Division and Polycystic Kidney Diseases

**DOI:** 10.3389/fcell.2020.578239

**Published:** 2020-09-22

**Authors:** Benjamin Vitre, Audrey Guesdon, Benedicte Delaval

**Affiliations:** CRBM, Univ Montpellier, CNRS, Montpellier, France

**Keywords:** microtubule – associated proteins, cell division, ciliopathies, molecular motor, intraflagellar transport

## Abstract

Cilia are small organelles present at the surface of most differentiated cells where they act as sensors for mechanical or biochemical stimuli. Cilia assembly and function require the Intraflagellar Transport (IFT) machinery, an intracellular transport system that functions in association with microtubules and motors. If IFT proteins have long been studied for their ciliary roles, recent evidences indicate that their functions are not restricted to the cilium. Indeed, IFT proteins are found outside the ciliary compartment where they are involved in a variety of cellular processes in association with non-ciliary motors. Recent works also provide evidence that non-ciliary roles of IFT proteins could be responsible for the development of ciliopathies related phenotypes including polycystic kidney diseases. In this review, we will discuss the interactions of IFT proteins with microtubules and motors as well as newly identified non-ciliary functions of IFT proteins, focusing on their roles in cell division. We will also discuss the potential contribution of non-ciliary IFT proteins functions to the etiology of kidney diseases.

## Introduction

Scientists have identified and described flagella since hundreds of years. However, the development of novel staining methods and the advance of microscopy techniques, in the second half of the 19th century allowed rapid progress in the description of this organelle. Importantly, those technological advances elicited the distinction between cilia and flagella and linked them to basal bodies and centrosomes (Bloodgood, 2009). In the second half of the 20th century, the use of transmission electron microscopy in cell biology revealed the presence of cilia in a variety of tissues and organisms. They organize in 9 + 2 and 9 + 0 tubular “fibrils,” the axonemes, emanating from basal bodies and extending longitudinally into cilia and flagella (Manton and Clarke, 1952; Fawcett and Porter, 1954; Barnes, 1961; Sorokin, 1962; Bloodgood, 2009). Those “fibrils” were later termed microtubules (Ledbetter and Porter, 1963; Slautterback, 1963). In the 1990’s and 2000’s, multiple studies linking cilia and cilia proteins to development and disease established the primary cilium as a central player in cell and tissue homeostasis.

First, the discovery of an intraflagellar transport (IFT) system within the flagellum of *Chlamydomonas reinhardtii* (Kozminski et al., 1993), its importance for cilia and flagella assembly (Cole et al., 1998; Pazour et al., 1998, 2000; Piperno et al., 1998) and the link between IFT proteins and ciliopathies attracted a great amount of attention on cilia (Pazour et al., 2000; Reiter and Leroux, 2017). IFT depends on three motors, homodimeric kinesin 2, heterotrimeric kinesin 2 and dynein 2 that move along axoneme microtubules. These motors carry IFT trains containing IFT proteins (Prevo et al., 2017). While the exact functions of the proteins involved in IFT are still being precisely studied, it is clearly established that they are essential for cilia formation and function (Reiter and Leroux, 2017). Second, the discovery that cilia can act as flow sensors (Praetorius and Spring, 2001, 2003) having a major impact on mammalian development asserted cilia as a central organelle for development (McGrath et al., 2003; Yoshiba et al., 2012; Ferreira et al., 2019). Third, the discovery that the IFT machinery and the primary cilium were involved in Sonic Hedgehog signaling, an essential developmental pathway associated with ciliopathy related phenotypes (Varjosalo and Taipale, 2008) and frequently upregulated in cancer, further raised attention on this organelle (Huangfu et al., 2003; Corbit et al., 2005). Later works also linked additional signaling pathways, such as wnt or notch, to cilia (Liu et al., 2018).

Driven by the identification of those three essential aspects, many studies have since been performed in order to understand cilia biogenesis and functions and their link to diseases. However, it also appears from this abundant literature that, when it comes to genes involved in ciliopathies, there is no simple relationship between mutations within a gene and the associated phenotypes (Reiter and Leroux, 2017). For example, mutations in IFT172 can result in a variety of phenotypes including retinal degeneration (retinis pigmentosa), obesity and polydactyly (Bardet Biedl Syndrome) (Bujakowska et al., 2015) or skeletal abnormalities, nephronophthisis and other clinical features associated with Jeune syndrome or Meinzer-Saldino syndrome (Halbritter et al., 2013). To explain this disconnection between mutations and phenotypes, it was proposed that stochastic events during the development of an individual harboring the mutation or that the genetic background of the individual could result in various phenotypes (Ben-Omran et al., 2015; Ramsbottom et al., 2015, 2020). As an alternative, it was also proposed that mutations in ciliary proteins could impair non-ciliary functions that may contribute to ciliopathies related phenotypes (Vertii et al., 2015; Hua and Ferland, 2018). Indeed, there is now increasing evidence that IFT proteins also function outside the cilium. IFT proteins are, for example, involved in the regulation of microtubule dynamics and/or organization in interphase cells (Bizet et al., 2015; Boehlke et al., 2015; Dupont et al., 2019). They are also required for specialized cellular functions such as the formation of the immune synapse in T cells (Finetti et al., 2009, 2014), the inflammatory response (McFie et al., 2020), the regulation of cell cycle progression (Robert et al., 2007) or cell division (Jonassen et al., 2008; Delaval et al., 2011; Borovina and Ciruna, 2013; Taulet et al., 2017, 2019; Vitre et al., 2020). We will summarize here our current knowledge on how IFT proteins interact and regulate microtubules and motors. We will review the current literature on their non-ciliary functions focusing on their roles in cell division. We will finally discuss how perturbations of the non-ciliary functions of IFT proteins could be relevant to cystic kidney diseases.

## Composition of IFT Trains in Cilia

**FIGURE 1:**
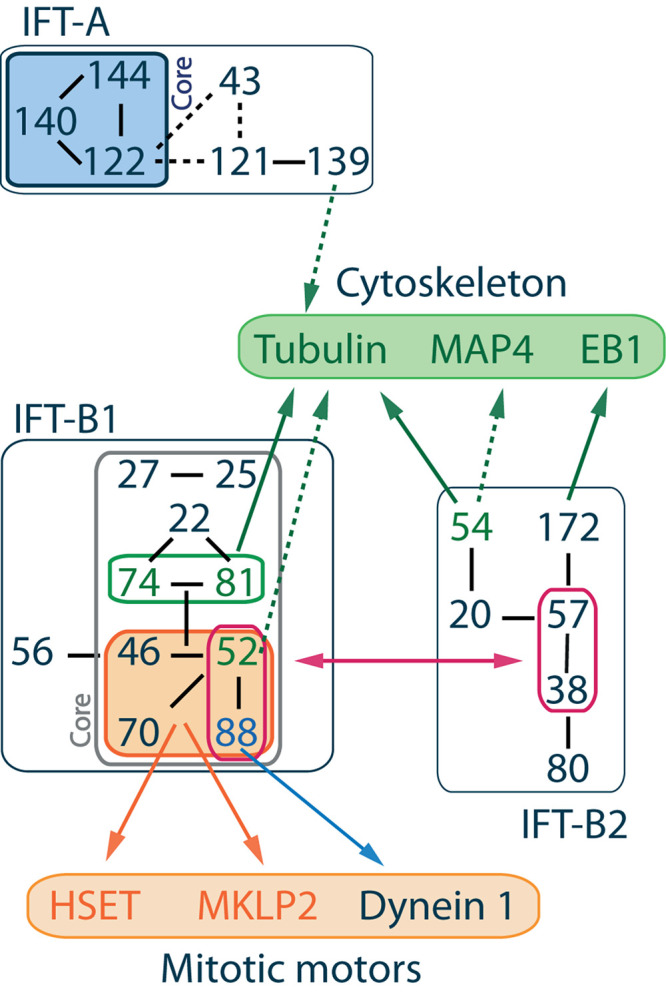
IFT complex overall organization and interactions with cytoskeleton components and mitotic motors. IFT proteins are divided into 2 classes. IFT-A complex is made of 6 subunits IFT144-140-139-122-121-43 where IFT144-140-122 form the core complex (Mukhopadhyay et al., 2010; Behal et al., 2012) and IFT139-120-43 form a peripheral subcomplex (Hirano et al., 2017). IFT-B complex is made of 16 subunits, and its overall architecture determined by VIP (visible immunoprecipitation) assays (Katoh et al., 2016), X-ray (Taschner et al., 2016), and interactome analysis (Boldt et al., 2016) reveals the presence of two stable subcomplexes. IFT-B1 is salt stable, its core complex is made of 9 subunits IFT88-81-74-70-52-46-27-25-22 (Lucker et al., 2005; Wang et al., 2009; Bhogaraju et al., 2011; Taschner et al., 2014; Wachter et al., 2019), while IFT-B2 is the peripheral complex made of 6 subunits IFT172-80-57-54-38-20 (Baker et al., 2003; Krock and Perkins, 2008; Taschner et al., 2016, 2018). The IFT-B2 central dimer IFT57-38 in association with the N-terminal domain of IFT52 and IFT88 mediates the interaction between both IFT-B subcomplexes (Taschner et al., 2016). Within IFT-B1, a stable tetrameric complex made of IFT88-70-52-46 was identified (Lucker et al., 2010; Taschner et al., 2011, 2014). Interactions between IFT machinery and mitotic motors are mediated by this tetramer (Delaval et al., 2011; Taulet et al., 2017; Vitre et al., 2020). Interactions with cytoskeleton components are mainly mediated by the IFT-B core complex, although the member of the IFT-A complex IFT139 is proposed to be a microtubule/tubulin binding partner (see text for details). Solid lines between IFT proteins indicate validated interactions, while dashed lines indicate putative interactions.

Cilia and flagella are evolutionarily conserved microtubule-based organelles found on eukaryotic cells. Since axonemal elongation occurs at the distal plus end of microtubules, ciliary assembly requires active transport of proteins from the base to the tip of the cilium along the axoneme. IFT corresponds to the continuous and bi-directional movement of large macromolecular assemblies under the ciliary membrane required for protein trafficking inside cilia (Iomini et al., 2001; Pedersen et al., 2005; Engel et al., 2009; Pigino et al., 2009; Buisson et al., 2013). Those particles, known as IFT trains, were first identified in the flagellum of *Chlamydomonas reinhardtii* by Differential Interference Contrast (DIC) microscopy (Kozminski et al., 1993). In this context, they were shown to mediate interactions between motors and cargoes that need to be delivered to the ciliary tip. Later studies showed that anterograde transport from the base to the ciliary tip is based on kinesin-2 motors (Walther et al., 1994; Kozminski et al., 1995; Cole et al., 1998), while retrograde transport toward the ciliary base is dependent on dynein-2 motors (Pazour et al., 1998, 1999; Perrone et al., 2003; Hou et al., 2004). Biochemical, structural and functional analysis of those complexes revealed several differences between anterograde and retrograde IFT complexes. Indeed, distinction was made between long and narrow IFT-A trains responsible for retrograde transport, and short and compact IFT-B trains responsible for anterograde transport (Cole et al., 1998; Stepanek and Pigino, 2016). IFT-A and IFT-B complexes differ in their molecular composition (Figure 1; Taschner and Lorentzen, 2016). Despite those differences, IFT proteins are highly conserved across species. Several protein-protein interaction motifs were identified as tetratricopeptide repeats, WD-40 repeats and coiled-coils that ensure the formation of IFT complexes and the interactions of IFT complexes with many cargoes and motors (Figure 1; Jékely and Arendt, 2006; Taschner et al., 2012).

## Ciliary and Non-Ciliary Interactions of IFT Proteins With Tubulin and Microtubules

Tubulin was shown to be an IFT cargo in *Chlamydomonas reinhardtii* (Craft et al., 2015) and *C. elegans* (Hao et al., 2011). Indeed, it is the most abundant ciliary protein that needs to be actively transported from the base to the tip of the cilium to ensure ciliary elongation. The interaction between soluble αβ-tubulin or polymerized microtubules and the IFT machinery is mediated by a highly conserved tubulin-binding module constituted by functional domains of IFT81 and IFT74 proteins. The first part of the module contains the N-terminal tubulin-binding calponin homology domain of IFT81 that recognizes αβ-tubulin dimers. The second part contains the highly basic N-terminal domain of IFT74 that interacts with the E-hook of β-tubulin and therefore increases the binding affinity of the module to tubulin dimers (Bhogaraju et al., 2013; Kubo et al., 2016; Weghe et al., 2020). However, the IFT74-IFT81 module has no ortholog in *Drosophila melanogaster* (van Dam et al., 2013; Bhogaraju et al., 2014). This suggests that other microtubule/tubulin binding sites might exist within IFT complexes. Indeed, IFT54/DYF-11/MIP-T3 was also proposed to be a binding partner of tubulin and actin (Guo et al., 2010). The binding activity with microtubule/tubulin was verified (Ling and Goeddel, 2000) and localized in the N-terminal part of IFT54/DYF-11/MIP-T3 (Taschner et al., 2016). This additional binding site could be responsible for the binding of two tubulin molecules per IFT-B complex. However, since IFT54 is not necessary for tubulin trafficking during ciliogenesis (Zhu et al., 2017), other microtubule/tubulin binding partners were proposed. IFT139 is for example able to pull down several ciliary precursors including tubulin (Qin et al., 2004). IFT57 and IFT38/DYF-3 also present a divergent calponin homology domain identified in several tubulin binding proteins (Schou et al., 2014; Taschner et al., 2016). However, the tubulin binding capacity of IFT57 and IFT38 have yet to be tested experimentally.

Through those interactions, IFT proteins can play regulatory roles on microtubules. As shown in *Chlamydomonas reinhardtii*, IFT proteins function as a tubulin transporter. They regulate microtubule polymerization and thus cilia growth, for example by concentrating soluble tubulin in cilia (Craft et al., 2015). Different studies also highlight the role of individual IFT proteins on the microtubule cytoskeleton. IFT70 is, for example, a regulator of ciliary tubulin polyglutamylation that is necessary for proper ciliogenesis in vertebrates (Pathak et al., 2007). IFT54 plays a role in the regulation of cytoplasmic microtubule dynamics by modulating the activity of MAP4, a microtubule-associated protein (MAP) stabilizing cytoplasmic microtubules (Bizet et al., 2015). IFT172 also interacts with the microtubule plus-end binding protein EB1 known to modulate microtubule dynamics and that localizes at the flagella tip (Pedersen et al., 2003, 2005). Altogether, this literature indicates that IFT proteins can play regulatory roles on the microtubule cytoskeleton both inside and outside the cilium by interacting with tubulin and microtubules directly or through MAPs.

## Non-Ciliary Functions of IFT Proteins in Dividing Cells

While most of the studies regarding IFT protein characterization were done in the context of cilia, it rapidly appeared that they could also function in non-ciliated dividing cells. Indeed, IFT52 localizes at the centrosomes constituting the mitotic spindle poles in *Chlamydomonas reinhardtii* (Deane et al., 2001). IFT74 localizes at the centrosome of mitotic human endothelial HUVEC cells (Iomini et al., 2004). IFT20 localizes at the Golgi and at the centrosome of mitotic RPE-1 cells (Follit et al., 2006). Finally, IFT88 also localizes at the centrosome of dividing RPE-1 and LLC-PK1 cells (Robert et al., 2007; Delaval et al., 2011).

**FIGURE 2:**
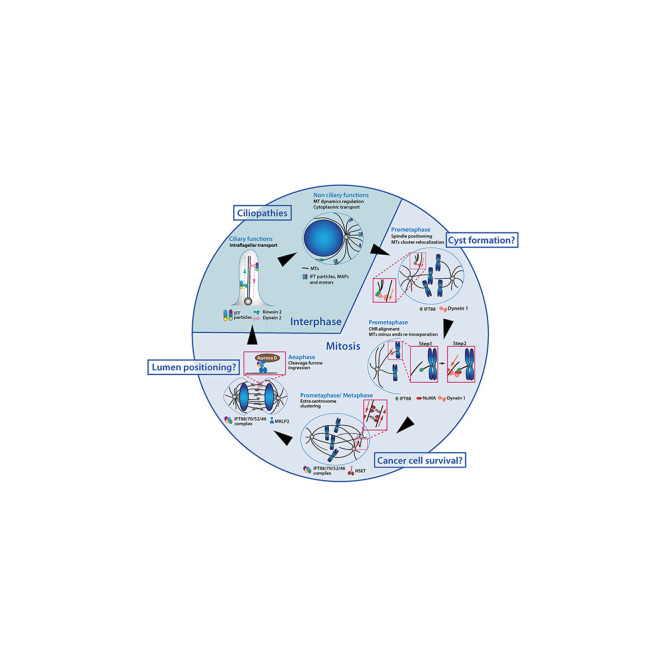
IFT proteins function throughout the cell cycle in association with microtubules and motors. IFT proteins contribute to a variety of interphase and mitotic molecular mechanisms. IFT proteins are required for ciliogenesis and for the regulation of cytoplasmic microtubules which were both linked to ciliopathies related phenotypes. Novel non-ciliary mechanisms for IFT proteins in mitosis have been identified. Perturbation of those mechanisms may also be linked to developmental defects and diseases. MTs, microtubules; CHR, chromosome.

In agreement with these localizations, IFT proteins were shown to contribute to various processes in dividing cells in association with microtubules and motors (Figure 2). Indeed, several works showed that IFT proteins contribute to proper mitotic spindle geometry and dynamics. IFT88 was initially described for its role in mitotic spindle orientation in kidney cells and zebrafish embryos (Delaval et al., 2011). In this context, IFT88, in association with the non-ciliary cytoplasmic dynein 1, is required for the re-localization of peripheral microtubule clusters to the poles of the mitotic spindle. This ensures efficient enrichment of astral microtubules and proper spindle orientation (Delaval et al., 2011). More recently, IFT88 was shown to control efficient mitotic spindle dynamics in prometaphase (Taulet et al., 2019). More specifically, IFT88 is required at minus end of kinetochore-bound-microtubules to recruit the microtubule binding protein NuMA (Nuclear mitotic apparatus protein) and cytoplasmic dynein 1. This recruitment ensures kinetochore fibers organization into spindle poles and proper chromosomes alignment (Hueschen et al., 2017). Finally, the IFT88-70-52-46 tetramer was shown to be necessary for efficient clustering of centrosomes during mitosis in cells harboring supernumerary centrosomes (Vitre et al., 2020). More specifically, the IFT proteins tetramer interacts directly with the mitotic motor HSET to ensure efficient centrosome clustering in mitosis. This mechanism is essential to ensure the proliferation and survival of cancer cells harboring supernumerary centrosomes. Of note, as opposed to this pro-survival role of IFT-B proteins in cancer cells with extra centrosomes, multiple studies have also shown that down-regulation of IFT88 drives pro-invasive phenotypes and cell proliferation. This was shown in hepatocellular carcinomas (Isfort et al., 1997; Huang Q. et al., 2017; Huang Y. et al., 2017; You et al., 2017) and in Hela cells (Robert et al., 2007).

Interestingly, several works also indicated that IFT proteins could function at later stages of mitosis to control cytokinesis. Indeed, several IFT proteins concentrate at the cleavage furrow in *Chlamydomonas reinhardtii* (Wood et al., 2012) and IFT88 was shown to control symmetrical cleavage furrow ingression during cytokinesis in mammalian kidney cells (Taulet et al., 2017). Mechanistically, the IFT88-70-52-46 complex directly interacts with the motor KIF20A/MKLP2 (Mitotic Kinesin-Like Protein 2). This is required for the timely and uniform relocalization of the Aurora B kinase toward the central spindle after anaphase onset, a mechanism that initiates cytokinetic furrow ingression. While IFT88 depletion does not prevent cytokinesis *per se*, this study showed that IFT proteins were required to control cell division geometry by controlling cleavage furrow ingression. It further showed that perturbations of cytokinesis geometry could have important detrimental consequences on 3D tissue organization.

Altogether those results unraveled key roles for IFT proteins in controlling cell division geometry in association with microtubules and motors.

## A Role for IFT Proteins in Ciliary and Non-Ciliary Motors Regulation?

Intraflagellar transport proteins have long been described as adapters between cargoes and motors. In cilia, proper movement of IFT complexes along the ciliary axoneme is mediated by interactions between IFT trains and molecular motors. Dynein-2 is responsible for the retrograde movement of IFT trains from the tip to the base of the cilia and needs to be transported to the ciliary tip as an inactive cargo via the anterograde IFT. This anterograde transport is mediated by kinesin motors. Kinesin-2 motors have different binding partners within the IFT-B complex. The kinesin-2 trimer KIF3A/KIF3B/KIFAP3 was shown to interact with the IFT88-52-57-38 tetramer (Funabashi et al., 2018). The coiled coil domain of IFT20 was identified as bridging KIF3B to IFT particles via its interaction with IFT57 (Baker et al., 2003; Krock and Perkins, 2008). The mammalian homodimeric kinesin-2 KIF17 was shown to directly interact with the IFT46-56 dimer and proposed to act as a cargo rather than a motor of IFT-B. This interaction is essential for its entry in the ciliary compartment (Howard et al., 2013; Funabashi et al., 2017).

However, the hypothesis that IFT subunits could directly impact and regulate the activity of ciliary motors was also proposed (Imanishi et al., 2006). In *C. elegans*, the IFT kinesin OSM3 exists in an autoinhibited form, and the binding of an IFT cargo, IFT70/DYF1 would relieve the autoinhibition of the motor. This hypothesis was recently confirmed *in vitro* (Mohamed et al., 2018). This work showed that OSM3 directly interacts with IFT70, leading to its activation. More specifically, IFT70 was shown to mediate the recruitment of the IFT-B sub-complex IFT88-70-52-46 to OSM3, triggering the full activation of the motor. This study demonstrated for the first time a direct regulatory role for IFT proteins on the activity of a ciliary kinesin.

Importantly, this work now raises the question of the potential impact of IFT proteins on the regulation and/or activation of other molecular motors. These include the recently characterized mitotic motors interacting with IFT proteins. However, whether IFT proteins could function as trafficking modules regulating motor activity and subsequently facilitating ciliary and non-ciliary transport will have to be addressed.

## Non-Ciliary Roles of IFT Proteins in Cystic Kidney Diseases

The various functions of IFT proteins outside cilia raised the question of their contribution to tissue homeostasis and diseases (Figure 2).

First clues in favor of a contribution of cell division defects induced by IFT proteins perturbations to kidney tissue disorganization came from the observation of abnormal cell division orientation in animal models of IFT depletion. Defects in spindle orientation were observed in IFT20 knock-out mice developing kidney cysts, although in this case the exact molecular mechanisms responsible for the spindle misorientation was not elucidated (Jonassen et al., 2008). Similarly, defects in mitotic spindle orientation were observed in the pronephric ducts of IFT88 depleted zebrafish embryos (Delaval et al., 2011). This suggested that IFT perturbations could contribute to kidney cyst initiation by controlling proper cell division orientation. The role of IFT88 in mitotic spindle orientation independently of ciliary defects was later confirmed *in vivo* in zebrafish embryos (Borovina and Ciruna, 2013). Of note, polycystic kidney diseases in animal models were also proposed to result from planar cell polarity (PCP) defects leading to abnormal cell division orientations (Fischer et al., 2006). Along this line, some genes involved in cystic kidney diseases appear to have no apparent function in ciliogenesis but to be necessary for cell division orientation and/or PCP pathways signaling. For example *seahorse*, a zebrafish cystic kidney gene, while being linked to cilia dependent Wnt signaling and PCP perturbation, does not appear to be involved in ciliary assembly (Kishimoto et al., 2008). Importantly, defects in spindle orientation following IFT depletion is not observed in all models of IFT perturbations suggesting that other non-ciliary mechanisms could be involved. Indeed, depletion of the IFT-A protein IFT140 in mice leads to kidney cyst formation but without evidence of mitotic spindle orientation defects (Jonassen et al., 2012). Here, it should be noted that developmental defects related to IFT-A protein depletion may also be due to Wnt signaling defects that are independent of the cilia as shown in non-ciliated drosophila epithelial cells (Balmer et al., 2015; Vuong et al., 2018).

A recent work also strengthened the link between cell division defects and cyst formation. Indeed, Taulet et al. (2017) who characterized the role of IFT88 in cytokinetic cleavage furrow ingression and also showed that perturbations of cytokinesis geometry lead to lumen mispositioning and subsequently to multifocal lumen formation in 3D culture of LLC-PK1 kidney cells. These defects are reminiscent of the multifocal lumen defects observed *in vivo*, in the early stages of polycystic kidney disease, in a mouse model carrying IFT88 mutation (Moyer et al., 1994).

Altogether these works from animal models indicate that the cell division defects induced by IFT proteins perturbations may contribute to cystic kidney diseases. At this stage, additional studies will be required to assess the non-ciliary contribution of IFT proteins to other developmental defects associated with ciliopathies. Interestingly, Peralta et al. (2020) recently demonstrated that IFT88 and IFT20 are involved in embryonic cardiogenesis in both mice and zebrafish. This developmental defect is due to a non-ciliary role of IFT proteins involving the Hippo pathway effector Yap1 outside the cilium, within the cytoplasm.

The contribution of cytoplasmic non-ciliary processes to disease also emerged from the study of human pathologies. Indeed, IFT54 mutations that are responsible for nephronophthisis, a progressive fibrosis of the kidney tubules ending with terminal renal failure, were shown to result in excessive MAP4 expression and microtubule stabilization in patient fibroblasts, while having limited effects on cilia (Bizet et al., 2015). In addition, introduction of those mutations in mouse kidney IMCD-3 3D cellular model results in epithelialization and polarity defects, phenotypes frequently observed in kidney diseases (Schlüter and Margolis, 2012). Similarly, increased α-tubulin acetylation, a posttranslational modification that is associated with stable and long lived microtubules (Piperno et al., 1987; Janke and Montagnac, 2017) was also found in human ARPKD cystic kidney samples and in cultured cells depleted of IFT88 (Berbari et al., 2013). While in this example ciliary defects occur concomitantly to the cytoplasmic microtubule phenotypes, it is worth noting that IFT proteins perturbation also affects cytoplasmic microtubules.

Finally, a recent study identified two mutations in IFT52 that cause short rib thoracic dysplasia and congenital anomaly of kidney and urinary tract. The authors showed, using mouse IMCD-3 cell culture, that these mutations perturb the interaction between IFT52 and centrin and trigger centriole splitting (Dupont et al., 2019). Additionally, they showed that IFT52 null IMCD-3 cells display defective cytoplasmic microtubule anchorage and dynamics. Of note, contrasting results were recently observed in human RPE-1 cells, where IFT52 depletion did not trigger centriole splitting in mitotic cells and did not affect microtubule dynamics in interphase cells (Vitre et al., 2020) indicating that the effects of IFT52 on those parameters could be cell type dependent.

Altogether, these works indicate that IFT proteins have microtubule-dependent functions outside the ciliary compartment. They also support the idea that non-ciliary functions may contribute, at least in part, to some developmental defects initially linked exclusively to cilia dysfunctions.

## Concluding Remarks

Overall, IFT proteins have to be considered as key regulators of ciliary and non-ciliary processes in association with microtubules and motors. Yet, we are only starting to understand the various roles these proteins may play beyond their role in cilia. Moreover, very little is known on the precise molecular mechanisms of these complexes as cargo and/or regulators of microtubules or ciliary and non-ciliary motors. Those regulatory functions of IFT proteins will have to be fully addressed, in the future, through *in vitro* reconstitution approaches. Finally, to further understand the exact contribution of non-ciliary functions of IFT proteins to ciliopathies it will now be essential to dynamically characterize the functional links between the non-ciliary functions of IFT proteins and 3D tissue architecture using 3D cell culture approaches and animal models.

## Author Contributions

BV, AG, and BD jointly wrote the manuscript. All authors contributed to the article and approved the submitted version.

## Conflict of Interest

The authors declare that the research was conducted in the absence of any commercial or financial relationships that could be construed as a potential conflict of interest.
